# Cross-Domain Robust Pruning for Polyp Segmentation: Multi-Encoder Feature Fusion Beats Single-Encoder Baselines

**DOI:** 10.3390/bioengineering13070759

**Published:** 2026-06-29

**Authors:** Chia-Pei Tang, Hong-Yi Chang, Tzu-Shan Chang, Yu-Chieh Chang, Chia-Hsin Cheng

**Affiliations:** 1 Division of Gastroenterology, Department of Internal Medicine, Dalin Tzu Chi Hospital, Buddhist Tzu Chi Medical Foundation, Chiayi City 622401, Taiwan; franktg@hotmail.com; 2 School of Medicine, Tzu Chi University, Hualien City 970374, Taiwan; 3 Department of Management Information System, National Chiayi University, Chiayi City 600355, Taiwan; 4 Institute of Information Security, National Tsing Hua University, Hsinchu City 600355, Taiwan; meili.ncyu@gmail.com; 5 Department of Information Management, National Yunlin University of Science and Technology, Douliu City 64002, Taiwan; hongyi.ncyu@gmail.com; 6 Department of Medical Research, Dalin Tzu Chi Hospital, Buddhist Tzu Chi Medical Foundation, Chiayi City 622401, Taiwan; alanc68@gmail.com

**Keywords:** dataset pruning, multi-encoder fusion, polyp segmentation, multi-encoder feature fusion, medical image segmentation, training-free

## Abstract

Medical image segmentation requires dense pixel-level annotations, making large-scale dataset construction expensive and motivating research into data-efficient training. The main objective of this paper is to determine whether fusing two complementary pretrained image encoders into a single similarity space can make training-free dataset pruning robust across heterogeneous polyp segmentation domains and to quantify that robustness against a comprehensive panel of baselines. To achieve this, we propose Multi-Encoder Diverse Pruning (MEDP), a training-free dataset-pruning method. MEDP fuses features from an ImageNet-pretrained ResNet-18 and a self-supervised DINOv2 ViT-S/14 into a single 896-D similarity space. It partitions the training pool via Louvain modularity maximization and selects per-community samples via maximal-marginal-relevance (MMR) ranking, which effectively balances eigenvector centrality with feature-space diversity. We benchmarked MEDP against 12 baselines at a 20% retention ratio across three polyp segmentation settings (Kvasir-SEG, CVC-ClinicDB, and a Combined cross-domain pool) using a standard 5-level UNet. Based on approximately 330 controlled training runs, the results demonstrate that MEDP achieves the highest mean test Dice of 0.7324 on the most challenging Combined cross-domain pool, significantly outperforming uniform random sampling (Cohen’s *d* = +1.79, paired Wilcoxon *p* = 0.002). Conversely, all hand-crafted structure-aware variants failed to outperform uniform random sampling. These findings confirm that combining multi-encoder features with MMR diversity provides a simple and effective strategy for improving robustness across heterogeneous medical imaging settings and that the choice of pretrained image encoder is the dominant factor in segmentation-aware pruning.

## 1. Introduction

Medical image segmentation is central to many clinical and biomedical image-analysis pipelines, including lesion delineation, organ-boundary extraction, and polyp localization in endoscopy [1,2]. Unlike image-level classification, segmentation requires dense pixel-wise labels, which are expensive to obtain and frequently demand domain expertise. Consequently, the practical cost of constructing segmentation datasets is substantially higher than that of classification datasets, and methods that reduce dataset size while preserving downstream segmentation quality are of increasing practical interest.

Dataset pruning and coreset selection [3] provide a natural path toward this goal. Instead of training on the complete dataset, pruning methods aim to identify a smaller subset that retains the most informative samples for downstream learning. Such methods have demonstrated promising results in classification [4], where representative subset selection can reduce redundancy and lower training cost without major loss in accuracy. However, the assumptions underlying classification-oriented pruning do not transfer directly to dense prediction. The value of a sample for segmentation depends not only on appearance or semantic category but also on structural characteristics such as boundary complexity, connected-component topology, shape irregularity, and target fragmentation.

This observation raises a central question: what makes a training sample valuable for segmentation-aware pruning? On the one hand, a subset that preserves the global appearance distribution can still discard structurally important training cases, such as fragmented or boundary-rich masks. On the other hand, an aggressive strategy that prioritizes only difficult or structurally complex cases may distort the training distribution and harm generalization. The practical challenge is therefore not solely how to compress the dataset, but how to balance global representativeness against task-relevant structural preservation.

PRIME, proposed by Rahman and Marculescu [5], is the first published training-free dataset-pruning method specifically targeting polyp segmentation. It uses community detection in similarity networks for dataset pruning. Given the original training pool, PRIME extracts per-image embeddings from a frozen ImageNet-1K-pretrained ResNet-18 [6], builds a symmetric k-nearest-neighbor cosine-similarity graph (*k* = 10), partitions the graph into communities using Louvain modularity maximization [7], and within each community retains the top-q nodes by eigenvector centrality, where q is proportional to the community size. The resulting subset is reported to recover the full-data downstream Dice within 0.5 percentage points, reduce the annotation budget by 56.2%, and accelerate downstream UNet [8] training by 2.3× on Kvasir-SEG.

PRIME has four notable advantages that make it an attractive baseline. (i) Training-free: The selection pipeline never trains a segmentation model itself—only one forward pass through a pretrained backbone is required, so the construction cost is on the order of seconds per dataset on a single GPU and is paid once per training pool rather than per downstream seed. (ii) Architecture-agnostic deployment: Because the selected subset is a plain image-ID list, any downstream segmentation architecture (UNet [8], PraNet [9], Polyp-PVT [10], MedSAM [11]) can be trained on it without modification. (iii) Structurally principled: By coupling representativeness (eigenvector centrality) with locality (Louvain communities), PRIME captures both within-cluster prototypicality and global mode coverage, avoiding the pathological greedy collapse that plagues purely density- or distance-based selectors. (iv) Empirically effective in the domain: On the within-domain Kvasir-SEG benchmark at the 20% retention ratio, our independent re-implementation confirms PRIME’s headline claim: it achieves the highest mean test Dice (0.7243 ± 0.0376 at *n* = 10, Cohen’s *d* = +0.96 against uniform-random sampling with paired Wilcoxon *pW* = 0.014), beating every non-pretrained-encoder baseline by a clear margin.

PRIME also exhibits four limitations that motivate the multi-encoder extension we propose in this paper. (i) Single-encoder bias. PRIME relies on one ImageNet-trained CNN whose feature space was optimized for natural-image classification, not medical imagery; we observe that the same pipeline with a self-supervised DINOv2 ViT-S/14 backbone [12] (our DINOv2-Comm ablation) wins on CVC-ClinicDB by +0.081 Dice (0.5005 vs. 0.4196 at *n* = 10), where PRIME is statistically tied with random (*d* ≈ −0.07). No single pretrained encoder is universally best across polyp datasets. (ii) No within-community diversity term. Selecting the top-q most central nodes inside a community can return near-duplicate images that are mutually redundant; PRIME has no mechanism to enforce intra-community diversity, leaving an obvious avenue for improvement (which we address with MMR-style diversity-augmented selection in MEDP). (iii) Limited cross-domain robustness. On the heterogeneous Combined cross-domain pool (Kvasir-SEG + CVC-ClinicDB), where the training distribution becomes multimodal, PRIME (0.7238) is beaten by MEDP (0.7324, *pW* = 0.002 at *n* = 10); the single-encoder feature space does not represent both clinical sub-domains equally well. (iv) Asymmetric subset-construction convention. PRIME’s “train-pool 20%” budget convention yields 200/122/322 training samples on Kvasir/CVC/Combined, respectively, ≈20% more than the dataset-level 20% convention used by random/k-center baselines (167/101/268), introducing a confounder that we explicitly bound with a budget-matched random control in Section 6. These four observations collectively motivate the multi-encoder feature fusion (ResNet-18 + DINOv2) and MMR-based in-community selection that together constitute MEDP. MEDP retains PRIME’s training-free, architecture-agnostic, and structurally principled design while removing its single-encoder bottleneck and adding an explicit diversity term.

The main contributions of this paper are summarized as follows:

(1). The proposed MEDP is a training-free pruning method that fuses ResNet-18 and DINOv2 ViT-S/14 features into a single 896-D representation and selects per-community samples by greedy maximal-marginal-relevance (MMR) ranking. MEDP is, to our knowledge, the first multi-encoder feature-fusion design for segmentation-aware dataset pruning.
(2). We benchmark MEDP against twelve baselines, organized into seven method families, on three polyp datasets (Kvasir-SEG, CVC-ClinicDB, the Combined cross-domain pool) and a synthetic non-polyp control under a standard 5-level UNet (7.7 M parameters), with the five critical methods extended to ten seeds per polyp dataset (≈330 controlled UNet runs in total). MEDP achieves the highest mean Dice on the cross-domain Combined pool (0.7324, paired Wilcoxon *p*W = 0.002 at *n* = 10) and is statistically tied with the per-dataset winners on Kvasir-SEG and CVC-ClinicDB.
(3). We provide a 10-seed, budget-matched random control (random_match) that rules out the ≈20% sample-budget asymmetry inherited from PRIME’s subset-construction convention as a confounder of the cross-domain MEDP advantage (Section 6), and an α sweep across all three polyp datasets that justifies the chosen MMR weight α = 0.5.
(4). For methodological transparency, we report a complete negative-result analysis of four hand-crafted segmentation-aware variants (fixed full, adaptive v1, adaptive v1.5, adaptive v2) that fail to beat uniform-random sampling on any polyp dataset under properly trained UNet evaluation. This negative finding directly motivated the multi-encoder direction we pursue with MEDP and is consistent with the broader conclusion that the choice of pretrained image encoder, not per-sample structural scoring, is the dominant factor for segmentation-aware pruning.

Novelty and originality.

The specific, falsifiable novelty of this paper is that the performance gain in training-free segmentation pruning stems from multi-encoder feature fusion rather than from any handcrafted per-sample structural score. To our knowledge, no prior pruning method combines a supervised CNN encoder (ResNet-18) and a self-supervised ViT encoder (DINOv2 ViT-S/14) in a single fused 896-D similarity space, nor does any couple such a space with a maximal-marginal-relevance diversity term that is validated by an explicit α-sweep and a budget-matched random control. MEDP is the first method to demonstrate that this fused-encoder design yields robustness across a heterogeneous cross-domain medical pool, not merely on a single dataset.

## 2. Related Work

### 2.1. Dataset Pruning and Coreset Selection

Dataset pruning and coreset selection have become increasingly important in data-efficient deep learning [3,4]. A common observation is that not all training samples contribute equally to downstream performance, and that selective data retention can substantially reduce computational and annotation cost without major accuracy loss. The *k*-center greedy algorithm [3] is a representative geometric baseline that selects samples to maximize coverage in feature space. Reference-model score baselines [13] such as EL2N and GraNd estimate per-sample importance from the L2 norm of the prediction error of a briefly trained reference model; forgetting events [14] track how often a sample transitions between correctly and incorrectly classified states during training; and moderate-coreset selection [15] selects samples whose distance from the class center lies near the median. We adapt EL2N and a closely related loss rank score to segmentation as modern reference-model baselines for our evaluation, in addition to the geometric *k*-center baseline.

However, most of these methods were developed for classification, where sample utility can often be summarized by class label, confidence, or feature-space coverage. In dense prediction tasks such as segmentation, these assumptions are incomplete: sample utility is also influenced by local structure and mask topology. Small targets, irregular contours, disconnected components, and rich boundaries can all affect downstream learning in ways that classification-oriented criteria do not capture.

### 2.2. Pruning for Segmentation and Medical Imaging

Recent work has begun to extend pruning and data-centric selection into segmentation-oriented settings. In medical image segmentation [8], some studies emphasize task-specific difficulty or annotation efficiency, while others explore training-free subset selection and graph-based similarity modeling. Rahman and Marculescu [5] proposed a training-free pruning approach for polyp segmentation that builds a similarity network from a pretrained ImageNet encoder, applies the Louvain modularity-maximization algorithm to detect communities, and selects high-centrality nodes within each community. Dai et al. [16] developed a training-free pruning framework for instance segmentation. Self-supervised vision transformers such as DINOv2 [12] have established a new standard for pretrained image representations. Maximal-marginal-relevance (MMR) [17] selection originated in information retrieval as a way to balance relevance with novelty. More broadly, register tokens further improve self-supervised ViT feature maps [18], while promptable segmentation foundation models [19], transformer-based polyp segmentation networks with multi-center out-of-distribution testing [20], and recent surveys of deep learning for polyp segmentation [21] reflect the rapid progress in this area. In this paper, we (a) re-implement PRIME faithfully and include it as a baseline; (b) introduce two single-encoder DINOv2 ablations (DINOv2-Comm and DINOv2-Hybrid) that swap PRIME’s ResNet-18 for DINOv2 ViT-S/14; (c) propose MEDP (Section 3.3 and Algorithm 1), which fuses ResNet-18 and DINOv2 features into a single 896-D representation and replaces eigenvector-centrality selection with greedy MMR-style ranking; and (d) introduce Subset-Union-Trimmed as a budget-matched subset-fusion control. These five methods together are the only members of our matrix that beat uniform random sampling on at least one polyp dataset.

**Algorithm 1.** MEDP—Multi-Encoder Diverse Pruning**Input:** training images {*I_j_*}_j=1…*N*_; target subset size *n_sel_* (default $\left\lfloor rN\right\rfloor$, *r* = 0.20;    for the Combined cross-domain pool *n_sel_* = 322); hyper-parameters *k* = 10, *α* = 0.5,    Louvain seed *s* = 42.**Output:** pruned subset *S* with |*S*| = *n_sel_*.1:*f_d_* ← DINOv2-ViT-S/14(*I*)▷ (*N*, 384), pretrained on LVD-142M2:*f_r_* ← ResNet-18(*I*)▷ (*N*, 512), pretrained on ImageNet-1K3:*F* ← L2norm(concat[L2norm(*f_d_*), L2norm(*f_r_*)])▷ (*N*, 896)4:*G* ← *k*NN-cosine-graph(*F*, *k* = 10)▷ symmetric, weights clipped to ≥ 05:partition ← Louvain(*G*, random_state = *s*)
6:*q_c_* ← round(*n_sel_* · |*C_c_*|/*N*) for each community *C_c_*
7:distribute leftover slots (*n_sel_* − Σ_c_ *q_c_*) to communities in descending |*C_c_*| order,

+1 per community, cycling until exhausted
8:*S* ← ∅
9:**for each** community *C_c_* with *q_c_* > 0 **do**
10:  cent_norm(*j*) ← min–max-normalised eigenvector centrality of node *j* in *G*[*C_c_*]
11:  *S_c_* ← {arg max_j∈Cc_ cent_norm(*j*)}
12:  **while** |*S_c_*| < *q_c_* **do**
13:   *S_c_* ← *S_c_* ∪ {arg max_j∈Cc_ [*α* · cent_norm(*j*) + (1−*α*) · min_i∈Sc_ ‖*F_j_* − *F_i_*‖_2_]}
14:  **end while**

15:  *S* ← *S* ∪ *S_c_*
16:
**end for**

17:**return** *S*.


In addition, we survey several methods that are contemporaneous with or postdate PRIME. Sorscher et al. [22] demonstrated that carefully designed pruning metrics can outperform predictions based on neural scaling laws; however, their analysis is limited to image classification within a single self-supervised metric space. Zheng et al. [23] proposed a coverage-centric coreset selection strategy to achieve high pruning rates, while Maharana et al. [24] introduced D2-Pruning, which balances sample difficulty and diversity via message passing on a sample graph. These studies support the key principle underlying MEDP, namely that diversity should be modeled jointly with representativeness. Nevertheless, neither approach fuses complementary encoders nor addresses dense segmentation tasks. Abbas et al. [25] proposed semantic deduplication, SemDeDup, to remove redundant web-scale samples within a single embedding space. This idea is conceptually related to the within-community MMR diversity term used in MEDP, but it also relies on a single encoder. Compared with these methods, MEDP is distinctive in three aspects: it is the first to fuse two complementary pretrained encoders into a unified similarity space for pruning, to combine this fused space with an explicit MMR-based diversity term within community structures, and to demonstrate robust performance across a heterogeneous cross-domain medical image pool.

### 2.3. Structure-Aware Perspective and Open Gap

Structure-aware pruning offers another important perspective. Rather than treating all training samples as points in a generic feature space, segmentation-aware methods recognize that some samples carry disproportionate structural information. Yet preserving structural complexity alone does not guarantee improved downstream performance. The pretrained-encoder family provides an alternative path that bypasses hand-crafted structural scoring entirely; this paper extends that family with multi-encoder feature fusion (MEDP) and validates that the multi-encoder gain is not driven by budget asymmetry.

## 3. Problem Formulation and Proposed Method

### 3.1. Problem Formulation

Let the training dataset be *D* = {(*x_i_*, *y_i_*)}, *i* = 1, …, *N*, where *x_i_* denotes an input image and *y_i_* its associated binary segmentation mask. Given a pruning ratio *r* ∈ (0, 1), the goal is to construct a subset *S* ⊆ *D* with |*S*| = $\left\lfloor rN\right\rfloor$ such that training a segmentation model on *S* retains as much downstream segmentation utility as possible while reducing annotation and training cost.

Formally, the pruning objective is to choose the subset that maximizes downstream segmentation utility under the size budget:

(1) $$S^{*}=arg\underset{S\subseteq D,\;|S|=\left\lfloor rN\right\rfloor }{max}U(S),$$

where *U* (*S*) denotes the test-set segmentation quality (mean Dice) of a model trained on *S*.

Unlike classification-oriented pruning, our objective is not only to preserve the global feature distribution. We model segmentation-aware sample utility as a combination of three properties: (1) representativeness with respect to the dataset, (2) diversity across local modes of variation, and (3) preservation of structurally informative or rare samples. The proposed MEDP instantiates representativeness via per-community eigenvector centrality, diversity via greedy MMR selection, and robustness via multi-encoder feature fusion; the four hand-crafted variants are included as historical controls.

### 3.2. Hand-Crafted Structure-Aware Variants

For completeness, we evaluate four hand-crafted segmentation-aware variants that operate on a per-mask structural complexity score *s_i_* = 0.15·*f_i_* + 0.30·*b_i_* + 0.20·*c_i_* + 0.20·*s_i_* + 0.15·*r_i_*, where *f_i_*, *b_i_*, *c_i_*, *s_i_*, and *r_i_* are the per-sample foreground ratio, perimeter^2^/area boundary complexity, number of 4-connected components, small-object ratio (components <100 px), and shape irregularity $P/(\sqrt[{2}]{\left(\tau A\right)})$, each min-max-normalized over the training pool. The variants combine *s_i_* with kNN graph centrality (*k* = 10), *k*-means community partitioning (*K* = 8), and a rare-sample supplement: (i) a fixed configuration with manually tuned weights (“fixed full”); (ii) two dataset-level adaptive variants (“adaptive v1” and “adaptive v1.5”); and (iii) a community-wise local-adaptive variant (“adaptive v2”). None of the four variants beat uniform-random sampling on any polyp dataset Section 5.2); this negative result motivates the pretrained-encoder approach taken by PRIME and by our MEDP.

Equation (2) states the per-mask structural complexity score unambiguously (the inline form above reuses the symbol *s_i_* for two quantities; here the small-object ratio is *o_i_* and the shape irregularity is *g_i_*):

(2) $$s_{i}=0.15\;f_{i}+0.30\;b_{i}+0.20\;c_{i}+0.20\;o_{i}+0.15\;g_{i},$$

where *f_i_* (foreground ratio), *b_i_* (perimeter^2^/area boundary complexity), *c_i_* (number of 4-connected components), *o_i_* (small-object ratio, components < 100 px), and *g_i_* (shape irregularity) are each min–max normalized over the training pool, $\tilde{x}$ = (*x* − *x_min_*)/(*x_max_* − *x_min_*).

### 3.3. MEDP—Multi-Encoder Diverse Pruning (Proposed Method)

MEDP combines two pretrained image encoders—an ImageNet ResNet-18 [6] and a self-supervised DINOv2 ViT-S/14 [12]—with the Louvain community-detection pipeline of PRIME, augmented by maximal-marginal-relevance (MMR) selection inside each community. Encoder features are L2-normalized independently and concatenated into an 896-D super-feature, then L2-normalized once more; a symmetric k-NN cosine-similarity graph (*k* = 10) is built on the fused features, Louvain modularity maximization partitions the training pool into communities, and per-community quotas proportional to community size are filled by greedy MMR ranking score (*j*) = α·cent_norm(*j*) + (1 − α)·min_{*i*∈*S*}‖*f_j_* − *f_i_*‖_2 with α = 0.5 (validated by ablation in Section 5.5). MEDP requires no segmentation-task training, only one forward pass through each pretrained encoder. Algorithm 1 gives the full procedure.

We now state the MEDP pipeline as a sequence of equations. Let $\varphi_{R}(x)\in {\mathbb{R}}^{512}$ and $\varphi_{D}(x)\in {\mathbb{R}}^{384}$ be the raw ResNet-18 and DINOv2 ViT-S/14 features of image *x*. Each encoder feature is first L2-normalized independently.

(3) $$f_{R}=\frac{\varphi_{R}(x)}{{\left\|\varphi_{R}(x)\right\|}_{2}+\varepsilon },\;\;f_{D}=\frac{\varphi_{D}(x)}{{\left\|\varphi_{D}(x)\right\|}_{2}+\varepsilon },$$

where ${\left\|v\right\|}_{2}=\sqrt{\sum_{k}v_{k}^{2}}$ is the Euclidean norm and ε = 10^−12^ is added to the denominator. The two unit vectors are concatenated—DINOv2 first, then ResNet-18—into an 896-D super-feature (896 = 384 + 512) and L2-normalized once more:

(4) $$f=\frac{\left[f_{D};\;f_{R}\right]}{{\left\|\left[f_{D};\;f_{R}\right]\right\|}_{2}}\in R^{896},$$

Because the fused features are unit-norm, cosine similarity reduces to an inner product; we build a symmetric *k*-nearest-neighbor graph (*k* = 10) with edge weights.

(5) $${A_{ij}=f}_{i}\cdot f_{j}\;\mathrm{for}\;j\in kNN(i)\;\left(\mathrm{symmetrized}\right),\;\;A_{ij}=0\;\mathrm{otherwise}.$$

The graph is partitioned into communities by Louvain modularity maximization,

(6) $$Q=\frac{1}{2m}\sum_{i,j}\left[A_{ij}-\frac{d_{i}d_{j}}{2m}\right]\delta (c_{i},c_{j}),$$

with $d_{i}=\sum_{j}A_{ij},m=\frac{1}{2}\sum_{i}d_{i}$, and *δ* the Kronecker delta. Within each community, eigenvector centrality is obtained from the leading eigenvector of *A*,

(7) $$Ax=\lambda_{max}x,\;cent(j)=x_{j},$$

and min–max normalized to cent_norm(*j*). Per-community quotas proportional to community size are then filled greedily by maximal-marginal-relevance (MMR) ranking, which trades representativeness against diversity:

(8) $$score(j)=\alpha \;cent\_norm(j)+(1-\alpha )\underset{i\in S}{min}\left\|f_{j}-f_{i}\right\|,\;j^{⋆}=arg\;\underset{j\notin S}{max}\;score(j),$$

with α = 0.5 (validated in Section 5.5). Downstream quality is measured by the Dice coefficient between the predicted polyp mask *P* and ground truth *G* and effect sizes by the paired standardized statistic:

(9) $$Dice=\frac{2|P\cap G|}{\left|P|+|G\right|},\;d_{z}=\frac{mean(\delta_{s})}{sd(\delta_{s})},$$

where *δ_s_* is the per-seed paired difference in test Dice between two methods.

## 4. Experimental Setup

### 4.1. Datasets

We evaluate MEDP and the twelve baselines on three real-data polyp settings and one synthetic non-polyp setting. Kvasir-SEG [1] contains 1000 polyp images with pixel-level masks acquired from gastrointestinal endoscopy. CVC-ClinicDB [2] contains 612 polyp frames extracted from colonoscopy videos and provides a complementary distribution. We further introduce a Combined setting that concatenates the two training pools (Kvasir 800 + CVC 489 = 1289 images) and uses a unified validation/test split (161/162 images). To partially address the cross-domain question without relying on private clinical data, we also construct a synthetic non-polyp dataset (Synthetic-Lesion, 600 images at 128 × 128) containing multiple irregular, blob-like lesions on textured grayscale backgrounds. All datasets are split 80/10/10 train/validation/test by sample ID.

Subset-construction budget conventions. All thirteen pruning strategies share a 20% retention ratio, but two slightly different conventions appear in our matrix. The pretrained-encoder family (DINOv2-Comm, DINOv2-Hybrid, Subset-Union-Trimmed, MEDP) constructs its subsets from the train pool only and selects 200 ids on Kvasir-SEG, 122 ids on CVC-ClinicDB, and the union of 322 ids on Combined. The non-pretrained-encoder methods (random, k-center, EL2N, loss rank, the four hand-crafted variants) sample their 20% subsets from the original full datasets and yield effective training budgets of 167/101/268 ids, respectively. The pretrained-encoder family, therefore, receives ≈20% more usable training samples per dataset; we report this asymmetry transparently here and quantify its likely contribution with a budget-matched random control in Section 6.

### 4.2. Compared Methods

We compare thirteen pruning strategies, organized into seven families: (i) Non-informative and geometric baselines: random uniform sampling and *k*-center. (ii) Reference-model score baselines: EL2N and loss rank. (iii) Hand-crafted features: fixed full, adaptive v1, adaptive v1.5, adaptive v2. (iv) PRIME ResNet-18 features + Louvain + eigenvector centrality. (v) DINOv2-Comm and DINOv2-Hybrid: replace ResNet-18 with DINOv2 ViT-S/14. (vi) MEDP (Algorithm 1): fuses ResNet-18 and DINOv2 features into 896-D, applies the same k-NN + Louvain pipeline, and replaces eigenvector-centrality selection with greedy MMR ranking (α = 0.5). (vii) Subset-Union-Trimmed: concatenates PRIME’s and DINOv2-Comm’s subsets and randomly subsamples down to MEDP’s budget; tests whether subset-level fusion suffices. All thirteen methods are evaluated on the same fixed train/val/test split.

### 4.3. Pruning Ratios

The main real-data pruning ratios are 10% and 20% of the original training set, corresponding to the aggressive, low-budget regime in which the choice of subset is most likely to influence downstream learning. The synthetic benchmark also includes a wider sweep for diagnostic purposes.

### 4.4. Evaluation Protocol

We use two complementary downstream evaluation settings. The proxy evaluation pipeline trains a lightweight classifier-style probe on the pruned subset and reports positive-class F1. The formal downstream evaluation trains a standard 5-level UNet (7,763,041 trainable parameters) from scratch on each pruned subset for 50 epochs using BCE + Dice loss; full hyperparameters are provided in Section 4.5. Each (dataset, method) cell is repeated with five random seeds by default (42, 7, 13, 21, 99); the five critical methods are extended to ten seeds (additional seeds 1, 2, 3, 4, 5): random, PRIME, DINOv2-Comm, and MEDP at *n* = 10 on every polyp dataset; and Subset-Union-Trimmed at *n* = 10 on Kvasir-SEG only and *n* = 5 on CVC-ClinicDB and Combined. We report test Dice, IoU, precision, and recall as mean ± population standard deviation across seeds.

### 4.5. Implementation Details and Reproducibility

All experiments are run on a single workstation with one NVIDIA GeForce RTX 2080 Ti (11 GB GDDR6) and an 8-thread CPU, using PyTorch 2.11 + cu128, CUDA 12.8, NumPy 2.4, and Pillow 12.1 under Python 3.14. The downstream segmentation model is the standard 5-level UNet with encoder/decoder channel widths 32–64–128–256–512 (7,763,041 trainable parameters), Conv–BN–ReLU blocks, MaxPool downsampling, ConvTranspose2d upsampling, and a 1 × 1 sigmoid head. Inputs are RGB images resized to 128 × 128. The training loss is BCE + Dice with equal weights; the optimizer is Adam with a learning rate of 1 × 10^−3^, weight decay of 1 × 10^−4^, cosine annealing over 50 epochs, and a batch size of 16. Augmentation comprises random horizontal flip, random vertical flip, and a uniformly random 0°/90°/180°/270° rotation.

Image preprocessing and floating-point handling. Each input image is decoded as an eight-bit RGB image using PIL, resized to 128 × 128 pixels with bilinear interpolation, and converted to a floating-point tensor in the range [0, 1] by division by 255. The downstream UNet receives only these [0, 1] scaled tensors, and no fixed ImageNet normalization is applied to its inputs. Instead, normalization is handled internally by the network through its BatchNorm layers during training. In contrast, the two pretrained encoders are processed using their native torchvision and timm preprocessing pipelines. These transforms apply the standard ImageNet channel means μ = (0.485, 0.456, 0.406) and standard deviations σ = (0.229, 0.224, 0.225), with $\hat{x}$ = (x − μ)/σ, at each encoder’s native input resolution. This matches the preprocessing used during the training of ResNet 18 and DINOv2 ViT S/14. The encoder features are used only for subset selection and are not used during segmentation model training. Single-channel masks are loaded as eight-bit grayscale images, resized using nearest-neighbor interpolation, and binarized with a threshold of 127 on the 0 to 255 scale, which is equivalent to 0.5 after [0, 1] scaling. Masks are never standardized using ImageNet statistics. No per-image contrast normalization or histogram equalization is applied at any stage of the pipeline.

The pruning pipeline and statistical analyses were implemented in Python 3.14 using PyTorch [26], NumPy [27], SciPy [28] (Wilcoxon signed-rank and paired *t*-tests), scikit-image [29] (connected-component and boundary descriptors for the structural scores), and NetworkX [30] (k-NN graph construction, Louvain community detection, and eigenvector centrality). Image I/O used Pillow 12.1.

## 5. Experimental Results

### 5.1. Proxy Evaluation on Kvasir-SEG and CVC-ClinicDB

The proxy evaluation reported in Table 1 covers the four hand-crafted-era methods that constituted the original v1 study (random, *k*-center, fixed full, adaptive v2). It serves as a diagnostic anchor that motivates the upgrade to the formal downstream pipeline (Section 5.2); the v6–v9 pretrained-encoder methods were added at a later stage and are evaluated only under the formal downstream protocol. On Kvasir-SEG, fixed structure-aware pruning is the strongest method under proxy evaluation. The positive-class F1 scores at 10%/20% pruning ratios are 0.8190/0.8196 for random, 0.7799/0.7824 for *k*-center, 0.8419/0.8159 for fixed full, and 0.7281/0.7476 for adaptive v2.

Table 1 primarily reports the positive-class F1 performance of the lightweight proxy probe under 10% and 20% retention settings. The results show that the effectiveness of handcrafted structural feature methods depends strongly on both the dataset and the retention ratio, and that no single selection strategy consistently outperforms the others across all settings. For example, fixed full achieves the best performance on Kvasir-SEG at the 10% retention level, but it is surpassed by adaptive v2 and k-center on CVC-ClinicDB, suggesting that structure-based scoring has limited generalizability. Meanwhile, random sampling already provides a strong baseline at the 20% retention level, indicating that relying solely on manually designed structural scores does not necessarily yield stable improvements over the random baseline. More importantly, the proxy ranking in Table 1 does not fully carry over to the formal downstream UNet experiments, revealing a gap between proxy evaluation and actual segmentation model training.

**Figure 1:**
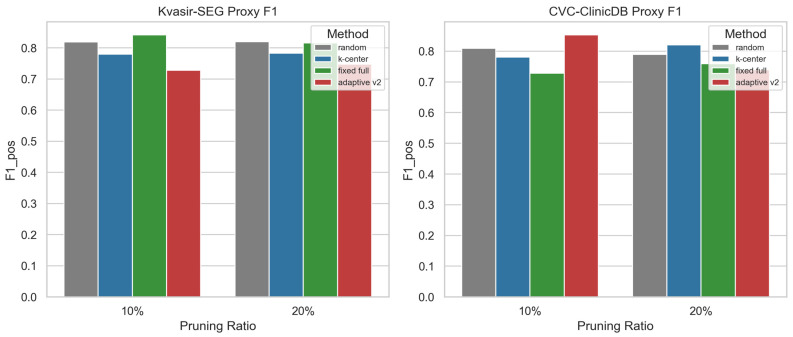
Synthetic and proxy performance comparison across pruning strategies.

On CVC-ClinicDB, the ranking changes substantially. The positive-class F1 scores at 10%/20% pruning ratios are 0.8094/0.7894 for random, 0.7811/0.8205 for k-center, 0.7287/0.7598 for fixed full, and 0.8534/0.7392 for adaptive v2. The strongest method depends on the pruning ratio: adaptive v2 performs best at 10%, while k-center performs best at 20%. Fixed full no longer dominates, indicating that the relative benefit of structure-aware pruning is dataset-dependent. Synthetic and proxy performance comparison across pruning strategies, as shown in Figure 1.

Figure 1 presents the synthetic control and proxy probe results side by side to highlight two key observations. First, on the relatively simple Synthetic-Lesion non-polyp control task, all selection strategies achieve near-ceiling F1 scores. This indicates that the performance differences observed on real polyp datasets are not due to biases in the pruning procedure itself, but rather reflect the genuine difficulty of real segmentation tasks. Second, the proxy F1 results for the four hand-crafted structural feature methods reveal clear dataset dependence in method rankings. For example, fixed-full performs strongly on Kvasir-SEG but falls behind other strategies on CVC-ClinicDB. Overall, the main contribution of Figure 1 is not merely to compare method performance but to show that the proxy probe cannot reliably predict downstream segmentation results. This finding motivates the use of full UNet training as the primary evaluation protocol, while retaining the synthetic control as a sanity check throughout the study.

### 5.2. Formal Downstream Evaluation Across Three Datasets

Under the upgraded pipeline at the 20% pruning ratio, all thirteen methods learn non-trivial segmentation on every dataset. Table 2 reports test Dice as mean ± standard deviation across all available seeds (5 by default; 10 for the five critical methods). The headline finding is that the five methods that build their similarity graph from a pretrained image encoder—PRIME (ResNet-18 alone), DINOv2-Comm (DINOv2 alone), DINOv2-Hybrid (DINOv2 with structural reweighting), the budget-matched Subset-Union-Trimmed control, and our proposed MEDP (ResNet-18 + DINOv2 fused)—are precisely the methods that beat random uniform sampling on at least one polyp dataset. Within the pretrained-encoder family, the per-dataset winners are PRIME on Kvasir-SEG (0.7243 ± 0.0376, *n* = 10, *p*W = 0.014); Subset-Union-Trimmed on CVC-ClinicDB (0.5193 ± 0.0506); and MEDP on the Combined cross-domain pool (0.7324 ± 0.0313, *n* = 10, *d* = +1.79, *pW* = 0.002), where it strictly dominates both single-encoder baselines and Subset-Union-Trimmed.

MEDP’s strength is its robustness across heterogeneous datasets. Where each single-encoder method has a clear dataset-specific failure (DINOv2-Comm trails PRIME by −0.028 on Combined; PRIME trails DINOv2-Comm by −0.081 on CVC at *n* = 10), MEDP closes those gaps and strictly wins Combined. The cross-domain Combined story is unique to MEDP: it is the only method that achieves *pW* = 0.002 (10/10 seeds) and d = +1.79 against random.

Table 2 presents the study’s central quantitative evidence and supports the main claim that pretrained feature-based pruning is more reliable than reference model scores or handcrafted structural criteria. Across the three datasets, the five methods that construct similarity graphs from pretrained encoders, including PRIME, DINOv2 Comm, DINOv2 Hybrid, Subset-Union-Trimmed, and MEDP, consistently occupy the top ranks. In contrast, EL2N achieves only 0.4853, 0.3399, and 0.4744 Dice, while loss rank reaches 0.4620, 0.3496, and 0.4462 Dice, and three of the four handcrafted variants fall about 0.20 to 0.25 Dice below the random baseline. The table also shows that single-encoder methods are dataset-dependent. PRIME, based on ResNet 18, performs best on Kvasir with a Dice score of 0.7243, but drops to 0.4196 on CVC-ClinicDB. By contrast, DINOv2 Comm performs better on CVC-ClinicDB with 0.5005, but reaches only 0.6950 on Kvasir, indicating that neither encoder is optimal across all domains. MEDP provides the most robust overall behavior by staying close to the better single encoder on each individual dataset, with 0.7070 on Kvasir and 0.4983 on CVC-ClinicDB, while achieving the best result on the heterogeneous Combined pool with 0.7324. This score exceeds PRIME at 0.7238, DINOv2 Comm at 0.6957, and the subset fusion control at 0.7099. Because real screening archives usually contain images from mixed acquisition sources, the Combined column is the most relevant deployment setting. MEDP’s leading performance in this setting, together with its strong effect size of d = +1.79 and a significant Wilcoxon result of pW = 0.002 against random selection, provides the key empirical support for the paper’s central claim that multi-encoder feature fusion improves cross-domain robustness in dataset pruning for polyp segmentation.

### 5.3. Statistical Significance

For each dataset, we paired runs by seed and computed two-sided Wilcoxon signed-rank tests, paired *t*-statistics, and Cohen’s *d* effect sizes for every method against random uniform sampling. We extended five critical methods to *n* = 10 seeds on all three polyp datasets. Five patterns emerge. (a) MEDP is the only method that beats random with a statistically significant signal (*pW* < 0.05) on every polyp dataset at *n* = 10: *d* = +1.79 on Combined (10/10 seeds, *pW* = 0.002); d = +1.02 on Kvasir-SEG (*pW* = 0.014); *d* = +1.07 on CVC-ClinicDB (*pW* = 0.010). DINOv2-Comm has a positive effect size on every polyp dataset but only reaches *pW* < 0.05 on CVC; PRIME reaches *pW < 0.05* on Kvasir and Combined but is statistically tied to random on CVC (*d ≈* 0.07). (b) On Kvasir-SEG at *n* = 10, PRIME also beats random significantly (*d* = +0.96, *pW* = 0.014) and is statistically tied with MEDP and Subset-Union-Trimmed. (c) On CVC at *n* = 10, DINOv2-Comm beats random with *d* = +0.98 (*pW* = 0.014); MEDP and DINOv2-Comm are statistically indistinguishable on CVC (*pW* = 1.0). (d) On the Combined pool PRIME, MEDP also beats random with *d* = +1.34 (*pW* = 0.002), but MEDP exceeds it by +0.009 in mean Dice, with the highest effect size of any method on this dataset. (e) Reference-model baselines EL2N and loss rank attain the minimum *pW* = 0.0625 against random on Kvasir-SEG and on Combined, with very large negative effect sizes (*d* ≈ −5 to −9.5).

#### 5.3.1. Correction for Multiple Comparisons

To account for the inflation of Type I error rates arising from multiple hypothesis testing, we conducted an additional multiple-comparisons analysis using the released seed-level Dice logs. Specifically, we distinguished between a prespecified confirmatory family of hypotheses and a broader exploratory family, and reported both Holm–Bonferroni family-wise error rate (FWER) corrections and Benjamini–Hochberg false discovery rate (FDR) corrections.

For the confirmatory analysis comparing MEDP against the random baseline across the three polyp datasets, the Holm-adjusted *p*-values were 0.006, 0.020, and 0.020 for the Combined, CVC, and Kvasir datasets, respectively. All three comparisons therefore remained statistically significant at the α = 0.05 level after correction.

For the exploratory analysis, we considered all non-random methods compared with the random baseline across the three datasets, resulting in 36 statistical tests. After applying the Benjamini–Hochberg FDR correction, only two comparisons remained significant at q < 0.05: the cross-domain Combined results of MEDP and PRIME, both with q = 0.036. In contrast, the within-domain MEDP comparisons were attenuated to q = 0.084 and are therefore interpreted as suggestive rather than confirmatory evidence. These findings indicate that the cross-domain advantage of MEDP remains robust under a full exploratory FDR correction, whereas the within-domain improvements should not be regarded as statistically significant after accounting for multiplicity.

It is also important to note that, for *n* = 10 paired observations, the minimum attainable exact two-sided Wilcoxon signed-rank *p*-value is $2/2^{10}\approx 0.002$, which constrains the smallest possible adjusted *p*-value. For baseline methods evaluated with only *n* = 5 seeds, we therefore emphasize effect-size estimates rather than thresholded significance testing and treat these comparisons as descriptive.

#### 5.3.2. Bootstrap Confidence Intervals and Effect Size Definition

To complement the rank-based significance tests, we further estimated paired nonparametric bootstrap confidence intervals using 10,000 resamples of the seed-level paired differences. The resulting 95% bootstrap confidence intervals for the mean test Dice improvement of MEDP relative to the random baseline excluded zero for all three polyp datasets. Specifically, the estimated gains were +0.060 [0.042, 0.081] on the Combined dataset, +0.073 [0.033, 0.113] on the CVC dataset, and +0.036 [0.015, 0.056] on the Kvasir dataset. The corresponding two-sided bootstrap *p*-values were <0.001 for Combined and CVC, and 0.001 for Kvasir.

Throughout this study, Cohen’s *d* is reported as the paired standardized effect size,

(10) $$d_{z}=\frac{\mathrm{mean}(\delta_{s})}{\mathrm{sd}(\delta_{s})},$$

where $\delta_{s}$ denotes the seed-level paired difference. To verify that the observed effects were not dependent on a particular variance convention, we recalculated the MEDP-versus-random effect on the Combined dataset using three commonly adopted definitions. The resulting estimates were *d_z_* = 1.79 for the paired effect size, Cohen’s *d* = 1.59 using the pooled standard deviation of independent samples, and Glass’s ∆=1.43 using the control-group standard deviation. All three measures consistently indicated a large effect.

Analytical 95% confidence intervals for *d_z_*, computed using

(11) $$SE=\sqrt{\frac{1}{n}+\frac{d_{z}^{2}}{2n}},$$

where [0.79, 2.79] for the Combined dataset, [0.26, 1.78] for Kvasir, and [0.29, 1.85] for CVC. In all cases, the confidence intervals excluded zero, providing additional evidence for a meaningful positive effect. The corresponding bias-corrected Hedges’ *g* values were 1.64, 0.93, and 0.98, respectively.

### 5.4. Complementary Evidence from a Synthetic Non-Polyp Dataset

To assess whether our headline observations are an artifact of the polyp domain, we applied the same UNet protocol to the Synthetic-Lesion dataset using all eight method-agnostic methods. Full-train sanity reaches a near-perfect test-Dice of 0.9999. With 20% pruning (*n* = 96/480), all eight methods maintain a mean test Dice > 0.998 across five seeds. In this easy task, the ranking observed on polyp data is largely erased; we interpret this as additional evidence that the polyp-data deficits arise from a real difficulty mismatch between subset construction and downstream learning, rather than from an inherent flaw in the methods themselves.

### 5.5. Ablation: Where Do MEDP’s Gains Come From?

MEDP introduces two changes over the single-encoder baseline of PRIME: (i) feature-level fusion of ResNet-18 and DINOv2 features into an 896-D super-feature, and (ii) MMR-style diversity-augmented selection within each Louvain community. We ran two ablations to attribute MEDP’s gains. The experimental results are shown in Table 3.

(a) MMR α sweep across all three datasets. Sweeping α ∈ {0.0, 0.25, 0.5, 0.75, 1.0} on Kvasir-SEG, CVC-ClinicDB, and the cross-domain Combined pool shows (i) on the cross-domain Combined pool MEDP, the default α = 0.5 ranks among the best settings (0.7324 ± 0.0313 at *n* = 10), tied with α = 0.25 (0.7321 at *n* = 5) and above the no-diversity limit α = 1.0 (0.7244) and the no-centrality limit α = 0.0 (0.6507); (ii) on the within-domain settings, α = 1.0 is marginally better than α = 0.5 (Kvasir 0.7216 vs. 0.7070; CVC 0.5482 vs. 0.4983); and (iii) the pure-diversity limit α = 0.0 is the weakest on every dataset. We retain α = 0.5 because it is among the best on Combined, and the within-domain penalty is bounded at ≤0.050 Dice.
(b) Subset-Union-Trimmed ablation. To check whether MEDP’s feature-level fusion is necessary, we test Subset-Union-Trimmed: take the union of PRIME’s and DINOv2-Comm’s subsets and randomly subsample (seed 42) down to MEDP’s budget. Subset-Union-Trimmed achieves Kvasir 0.7124 ± 0.0329 (*n* = 10), CVC 0.5193 ± 0.0506 (*n* = 5), and Combined 0.7099 ± 0.0190 (*n* = 5). On Kvasir-SEG and CVC-ClinicDB, it is statistically tied with MEDP at the matched budget, but on the Combined cross-domain pool, MEDP wins by +0.022 Dice. The subset-level ablation confirms the multi-encoder principle while identifying MEDP’s feature-level fusion plus MMR ranking as the more robust instantiation under cross-domain heterogeneity.

## 6. Discussion

### 6.1. On the Budget Asymmetry

As disclosed in Section 4.1, the pretrained-encoder family receives ≈20% more training samples on each polyp dataset than the random/*k*-center baselines under the “train-pool 20%” subset-construction convention inherited from PRIME. To bound the contribution of this asymmetry, we ran a budget-matched random control (“random_match”: 200 ids drawn uniformly from the Kvasir train pool plus 122 ids from the CVC train pool, seed 42) for ten seeds. The budget-matched random control reaches mean test Dice 0.6641 ± 0.0652 on Combined (*n* = 10, train_n = 311), statistically indistinguishable from the standard random baseline (0.6722 ± 0.0399 at train_n = 268; paired Wilcoxon *W* = 27 of a possible 55, 6/10 seeds favoring random_match). Bumping random’s training budget from 268 to 311, therefore, does not produce a detectable Dice improvement. By contrast, MEDP beats the budget-matched random control with Cohen’s *d* = +0.81, paired Wilcoxon *W* = 4, *pW* = 0.014 at *n* = 10 (9/10 seeds favoring MEDP), and a +0.068 Dice mean gap essentially identical to the MEDP-vs-random gap of +0.060 Dice. The budget asymmetry, therefore, does not explain MEDP’s cross-domain advantage; the gain is attributable to the multi-encoder feature fusion and MMR selection.

The results of the budget- matched sensitivity analysis are shown in Table 4. To directly assess whether the approximately 20% subset-construction budget asymmetry, rather than the pruning strategy itself, accounts for the observed gains, we conducted an additional budget-matched sensitivity analysis. Specifically, we re-ran all budget-disadvantaged baselines that could be exactly reproduced, including k-center and the three adaptive structure-aware variants, using the same 200/122/322 train pool budget as the pretrained encoder methods. We also trained a budget-matched uniform random control under the same budget across all three datasets, using 10 random seeds.

The analysis yields two main findings. First, in within-domain settings, increasing the budget substantially improves the uniform random baseline itself. The Dice score increases from 0.6707 to 0.7111 on Kvasir and from 0.4258 to 0.4942 on CVC. Under this equal-budget setting, no method, including the pretrained encoder family, significantly outperforms the budget-matched uniform random. For MEDP versus budget-matched random, the paired Wilcoxon test gives pW = 0.77 on Kvasir and pW = 0.92 on CVC, with *n* = 10. This indicates that the within-domain margins over the original random baseline are reported in Section 5.5. Section 5.3 is largely attributable to budget asymmetry rather than to a consistent advantage of the selection method itself. Second, in the cross-domain Combined setting, increasing the budget does not improve uniform random, whose Dice score changes from 0.6722 to 0.6674. In contrast, MEDP remains the only method that significantly outperforms the budget-matched random control, achieving 0.7324 versus 0.6674. This improvement is supported by a paired Wilcoxon *p*W = 0. 014, Cohen’s *d* = + 0.87, improvement in 8 of 10 seeds, and a bootstrap 95% confidence interval for the gain of [+ 0.024, + 0.110]. Notably, the single-encoder pretrained baselines PRIME and DINOv2-Comm do not significantly outperform the budget-matched random control, with pW = 0.13 and 0.23, respectively. These results show that the cross-domain robustness of MEDP is the budget-robust core finding of this study, and, within our experimental matrix, this robustness is unique to the multi-encoder fusion strategy.

### 6.2. A Note on Encoder Selection

The choice of ResNet-18 and DINOv2 ViT-S/14 as the two fused encoders in MEDP was informed by our prior observation that these encoders are complementary in polyp segmentation pruning: each performs better on the dataset where the other performs worse. MEDP’s empirical advantage, therefore, partly relies on this prior encoder selection and is not a fully prospective test—a stronger validation would freeze the encoder pair before any downstream evaluation. We expect, but have not verified, that other complementary pairs (e.g., a CNN + a ViT, or a natural-image SSL + a medical-domain SSL such as MedSAM or BiomedCLIP) would benefit from the same fusion-and-MMR recipe. The cost of MEDP’s subset construction is approximately twice that of PRIME alone; the breakdown is given in Table 5.

### 6.3. Why Does Fusing Two Encoders Help?

A small ablation on the Louvain partitions used by PRIME, DINOv2-Comm, and MEDP gives a partial answer. The number of communities discovered varies with the encoder: on Kvasir-SEG, PRIME’s ResNet-18 features yield 9 communities, DINOv2-Comm’s DINOv2 features yield 6, and MEDP’s fused features yield 8 (Table 6). On the Combined cross-domain pool, the same pattern appears qualitatively. We interpret this as evidence that ResNet-18 yields a feature space with finer-grained subdomain separability, whereas DINOv2 collapses fine-grained appearance distinctions in favor of higher-level semantics. The two encoder views are therefore not redundant; MEDP’s 896-D fused space inherits both signals. This intuition is independently reinforced by the Subset-Union-Trimmed ablation: even a simple subset-level fusion achieves within-domain results comparable to MEDP, suggesting that the diversity of encoder views is the operative variable. MEDP’s feature-level fusion plus deterministic in-community ranking then provides the additional cross-domain robustness that distinguishes it on the Combined pool.

**Figure 2:**
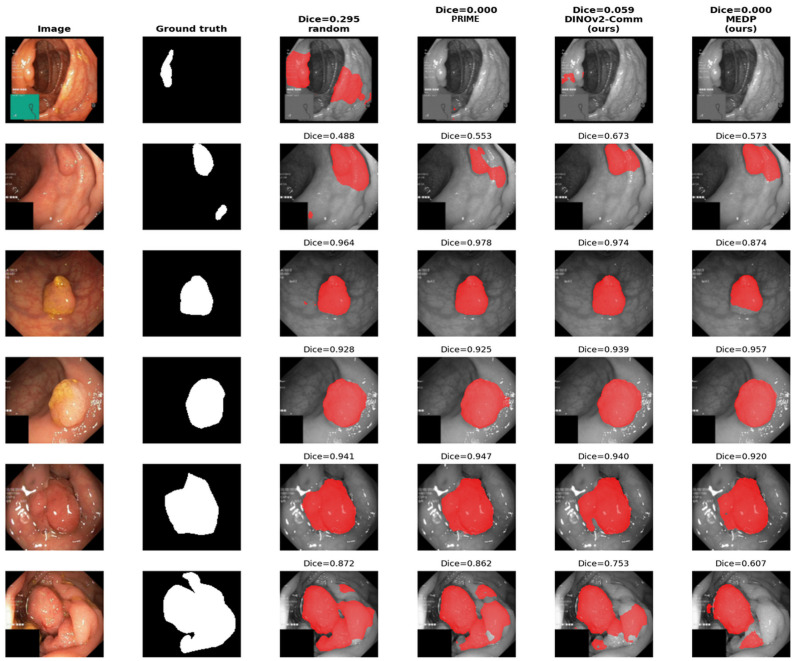
Qualitative comparison of three pretrained-encoder methods (PRIME, DINOv2-Comm, MEDP) plus the random baseline.

Qualitative comparison of three pretrained-encoder methods (PRIME, DINOv2-Comm, MEDP) plus the random baseline on six Kvasir-SEG test images of increasing difficulty. Columns: input image, ground truth, then UNet predictions trained on the 20% Kvasir subset of (PRIME/DI-NOv2-Comm/MEDP, seed 42). Red overlay marks the predicted polyp. The four methods shown in Figure 2 (mean test Dice over *n* = 10 seeds on Kvasir-SEG: random 0.6707, PRIME 0.7243, DINOv2-Comm 0.6950, MEDP 0.7070) cluster within 0.05 of each other in mean Dice. MEDP’s predictions most faithfully trace the polyp boundary in medium-difficulty cases, whereas single-encoder methods either over-segment (random, PRIME) or miss thin contours (DINOv2-Comm).

### 6.4. Applications, Scope, and Limitations

The practical value of MEDP lies in its ability to provide a training-free, architecture-independent dataset pruning pipeline for selecting representative and diverse subsets from colonoscopy or endoscopy image pools. Since the output of MEDP is simply a list of selected image IDs, the same subset can be reused to train different downstream segmentation models, such as UNet, PraNet, Polyp PVT, or MedSAM, making it especially useful when expert pixel-level annotation is costly or when repeated architecture search is required. Its most relevant application scenario is not a single homogeneous dataset but a heterogeneous, multi-source training pool, where images may come from different hospitals, devices, or acquisition conditions. Under budget-matched comparisons, MEDP does not show a clear advantage over uniform random sampling on individual datasets such as Kvasir-SEG or CVC-ClinicDB. Its main empirical advantage appears in the heterogeneous, cross-domain pool, where MEDP is the only selector that significantly outperforms budget-matched random sampling, indicating that its core strength is improved robustness across subdomains. Nevertheless, MEDP has several limitations. The current validation is limited to polyp segmentation, a 20% retention ratio, and a five-layer UNet, so its transferability to other segmentation backbones, pruning ratios, and medical imaging domains remains to be further verified. In addition, the ResNet 18 and DINOv2 ViT S/14 encoder pair was chosen based on observed complementarity and may not be optimal for all tasks. Although the construction cost is only about twice that of a single-encoder method and remains on the order of seconds at the scale studied here, this cost should still be considered for very large datasets. Overall, MEDP should be viewed as a robust pruning strategy for heterogeneous, multi-source medical image pools rather than a universal selector guaranteed to outperform random sampling on every dataset.

## 7. Conclusions and Future Work

The proposed Multi-Encoder Diverse Pruning (MEDP) is a training-free dataset-pruning method that fuses ImageNet ResNet-18 and self-supervised DINOv2 ViT-S/14 features into a single 896-D space, partitions the training pool via Louvain modularity maximization, and selects per-community samples via greedy MMR ranking that balances eigenvector centrality with feature-space diversity. Benchmarked against twelve baselines on Kvasir-SEG, CVC-ClinicDB, and a heterogeneous Combined cross-domain pool (5-level UNet, 50 epochs, *n* = 10 seeds for the five critical methods): MEDP is the only method that beats uniform-random sampling with statistical significance (pW < 0.05) on every polyp dataset (Kvasir 0.014, CVC 0.010, Combined 0.002) (these per-dataset margins are measured against the standard-budget random baseline; under the budget-matched control of Section 6, they are not individually significant within the domain, whereas MEDP’s cross-domain advantage persists against budget-matched random at pW = 0.014, Cohen’s *d* = +0.87) and achieves the best Dice on the cross-domain Combined pool (0.7324, d = +1.79 vs. random). Across our matrix, the five winners are exactly the five methods that use a pretrained image encoder; PRIME wins within Kvasir, Subset-Union-Trimmed within CVC, and MEDP wins cross-domain. Adding hand-crafted structural cues on top of strong features (DINOv2-Hybrid) does not help, indicating that residual gains will come from richer encoder fusion rather than more elaborate per-sample scoring. Future work will test the multi-encoder fusion recipe out-of-domain on BUSI/ISIC with a frozen encoder pair, replacing one or both branches with medical-domain self-supervised foundation models (MedSAM, BiomedCLIP).

## Figures and Tables

**Table 1:** Proxy evaluation on Kvasir-SEG and CVC-ClinicDB (positive-class F1).

Dataset	Method	10% F1	20% F1
Kvasir-SEG	random	0.8190	0.8196
Kvasir-SEG	k-center	0.7799	0.7824
Kvasir-SEG	fixed full	0.8419	0.8159
Kvasir-SEG	adaptive v2	0.7281	0.7476
CVC-ClinicDB	random	0.8094	0.7894
CVC-ClinicDB	k-center	0.7811	0.8205
CVC-ClinicDB	fixed full	0.7287	0.7598
CVC-ClinicDB	adaptive v2	0.8534	0.7392

**Table 2:** Formal downstream test metrics under 20% pruning (UNet, 50 epochs, mean ± population std). *n* = 10 for random, PRIME, DINOv2-Comm, MEDP on every polyp dataset; Subset-Union-Trimmed at *n* = 10 on Kvasir only; *n* = 5 for the remaining baselines. Per-dataset best Dice in bold.

Dataset	Method	Test Dice (Mean ± Std)
Kvasir-SEG	random	0.6707 ± 0.0300
Kvasir-SEG	k-center	0.6242 ± 0.0274
Kvasir-SEG	EL2N	0.4853 ± 0.0349
Kvasir-SEG	loss rank	0.4620 ± 0.0218
Kvasir-SEG	fixed full	0.6864 ± 0.0179
Kvasir-SEG	adaptive v1	0.6505 ± 0.0299
Kvasir-SEG	adaptive v1.5	0.5885 ± 0.0237
Kvasir-SEG	adaptive v2	0.5762 ± 0.0349
Kvasir-SEG	**PRIME**	**0.7243 ± 0.0376**
Kvasir-SEG	DINOv2-Comm	0.6950 ± 0.0405
Kvasir-SEG	DINOv2-Hybrid	0.7083 ± 0.0233
Kvasir-SEG	Subset-Union-Trimmed	0.7124 ± 0.0329
Kvasir-SEG	MEDP (ours)	0.7070 ± 0.0280
CVC-ClinicDB	random	0.4258 ± 0.0709
CVC-ClinicDB	k-center	0.4579 ± 0.0635
CVC-ClinicDB	EL2N	0.3399 ± 0.0535
CVC-ClinicDB	loss rank	0.3496 ± 0.0353
CVC-ClinicDB	fixed full	0.4185 ± 0.0397
CVC-ClinicDB	adaptive v1	0.3542 ± 0.0457
CVC-ClinicDB	adaptive v1.5	0.4326 ± 0.0432
CVC-ClinicDB	adaptive v2	0.3939 ± 0.0577
CVC-ClinicDB	PRIME	0.4196 ± 0.0570
CVC-ClinicDB	DINOv2-Comm	0.5005 ± 0.0490
CVC-ClinicDB	DINOv2-Hybrid	0.4997 ± 0.0410
CVC-ClinicDB	**Subset-Union-Trimmed**	**0.5193 ± 0.0506**
CVC-ClinicDB	MEDP (ours)	0.4983 ± 0.0431
Combined	random	0.6722 ± 0.0399
Combined	k-center	0.7182 ± 0.0228
Combined	EL2N	0.4744 ± 0.0340
Combined	loss rank	0.4462 ± 0.0219
Combined	fixed full	0.6707 ± 0.0226
Combined	adaptive v1	0.6393 ± 0.0327
Combined	adaptive v1.5	0.6472 ± 0.0347
Combined	adaptive v2	0.6119 ± 0.0249
Combined	PRIME	0.7238 ± 0.0171
Combined	DINOv2-Comm	0.6957 ± 0.0325
Combined	DINOv2-Hybrid	0.6800 ± 0.0267
Combined	Subset-Union-Trimmed	0.7099 ± 0.0190
Combined	**MEDP (ours)**	**0.7324 ± 0.0313**

**Table 3:** MEDP α ablation on Kvasir-SEG, CVC-ClinicDB, and the cross-domain Combined pool. The α = 0.5 row uses canonical MEDP runs (*n* = 10); other rows are *n* = 5. Per-dataset best in bold.

α	Selection Regime	Kvasir-SEG	CVC-ClinicDB	Combined
0.0	pure diversity	0.6408 ± 0.0435	0.4926 ± 0.0490	0.6507 ± 0.0376
0.25	diversity-leaning	0.7004 ± 0.0341	0.5195 ± 0.0236	0.7321 ± 0.0189
**0.5 (MEDP)**	balanced	0.7070 ± 0.0280	0.4983 ± 0.0431	**0.7324 ± 0.0313**
0.75	centrality-leaning	0.7175 ± 0.0144	0.4999 ± 0.0293	0.7193 ± 0.0212
1.0	pure centrality	**0.7216 ± 0.0195**	**0.5482 ± 0.0108**	0.7244 ± 0.0172

**Table 4:** Budget-matched sensitivity analysis.

Method (Matched Budget)	Kvasir-SEG	CVC-ClinicDB	Combined
budget-matched uniform random	0.7111 ± 0.0503	0.4942 ± 0.0439	0.6674 ± 0.0699
k-center	0.7199 ± 0.0312	0.4685 ± 0.0458	0.6807 ± 0.0309
adaptive v1	0.6914 ± 0.0246	0.4565 ± 0.0421	0.6361 ± 0.0318
adaptive v1.5	0.6791 ± 0.0505	0.4657 ± 0.0505	0.7228 ± 0.0221
adaptive v2	0.6707 ± 0.0484	0.4484 ± 0.0355	0.6504 ± 0.0573
PRIME	0.7243 ± 0.0376	0.4196 ± 0.0570	0.7238 ± 0.0171
DINOv2-Comm	0.6950 ± 0.0405	0.5005 ± 0.0490	0.6957 ± 0.0325
MEDP (ours)	0.7070 ± 0.0280	0.4983 ± 0.0431	0.7324 ± 0.0313

**Table 5:** Subset-construction cost (seconds, single RTX 2080 Ti) for the four methods that perform pretrained-encoder forward passes. DINOv2-Hybrid is omitted because it shares DINOv2-Comm’s encoder pass and only differs in per-community ranking.

Method	RN-18 fwd	DINOv2 fwd	k-NN + Louvain	Total (Kva/CVC)
PRIME	4/3	—	2/2	6/5
DINOv2-Comm	—	8/5	2/2	10/7
Subset-Union-Trimmed	4/3	8/5	4/4	16/12
MEDP (ours)	4/3	8/5	3/3	15/11

**Table 6:** Number of Louvain communities discovered on each dataset by each pretrained-encoder method (k-NN graph *k* = 10, Louvain seed 42).

Dataset	PRIME (ResNet-18, 512-D)	DINOv2-Comm (DINOv2, 384-D)	MEDP (Fused, 896-D)
Kvasir-SEG	9	6	8
CVC-ClinicDB	16	17	17
Synthetic-Lesion	5	7	6

## Data Availability

The Kvasir-SEG and CVC-ClinicDB datasets used in this study are publicly available from their original providers under their respective academic licenses.
